# Prevention of non-communicable diseases in Pakistan: an integrated partnership-based model

**DOI:** 10.1186/1478-4505-2-7

**Published:** 2004-09-13

**Authors:** Sania Nishtar

**Affiliations:** 1 Heartfile, 1-Park Road, Chak Shahzad, Islamabad, Pakistan

## Abstract

Development and implementation of non-communicable disease (NCD) prevention polices in the developing countries is a multidimensional challenge. This article highlights the evolution of a strategic approach in Pakistan. The model is evidence-based and encompasses a concerted and integrated approach to NCDs. It has been modelled to impact a set of indicators through the combination of a range of actions capitalizing on the strengths of a public-private partnership. The paper highlights the merits and limitations of this approach. The experience outlines a number of clear imperatives for fostering an enabling environment for integrated NCD prevention public health models, which involve roles played by a range of stakeholders. It also highlights the value that such partnership arrangements bring in facilitating the mission and mandates of ministries of health, international agencies with global health mandates, and the non-profit private sector. The experience is of relevance to developing countries that have NCD programs running and those that need to develop them. It provides an empirical basis for enhancing the performance of the health system by fostering partnerships within integrated evidence-based models and permits an analysis of health systems models built on shared responsibility for the purpose of providing sustainable health outcomes.

## Background

Non-communicable diseases exhort a considerable toll on individuals, societies and health systems [1,2]. Located in South Asia, Pakistan has a population of 150 million and a per-capita health expenditure of US $ 18 [3]. NCDs and injuries are amongst the top ten causes of mortality and morbidity in Pakistan [4]; estimates indicate that they account for approximately 25% of the total deaths within the country [5]. NCDs contribute significantly to adult mortality and morbidity and impose a heavy economic burden on individuals, societies and health systems [6]. In most cases, it is the economically productive workforce, which bears the brunt of these diseases. Existing population-based morbidity data on NCDs in Pakistan show that one in three adults over the age of 45 years suffers from high blood pressure [7]. The prevalence of diabetes is reported at 10% whereas 40% men and 12.5% women use tobacco in one form or the other [8,9]. Karachi reports one of the highest incidences of breast cancer for any Asian population [10]. In addition, estimates indicate that there are one million severely mentally ill and over 10 million individuals with neurotic mental illnesses within the country [11]. Furthermore, 1.4 million road traffic crashes were reported in the country in the year 1999; of these, 7000 resulted in fatalities [12].

Established evidence highlights the potential to limit NCD mortality and morbidity through appropriate public health strategies aimed at disease prevention, risk factor control and health promotion [13]. Addressing NCDs in a developing country such as Pakistan is a multidimensional challenge with implications at different levels and necessitates a two fold action. Firstly, lobbying for appropriate investments and policies to facilitate their inclusion in the development and health agenda [14], and secondly, developing scientifically valid, culturally appropriate and resource-sensitive models incorporating and integrating the multidisciplinary range of actions relevant for NCD prevention.

In Pakistan, the public-private tripartite partnership led by Heartfile (a non-profit NGO registered under the Societies Registration act of 1860 in Pakistan) and constituted additionally by the Ministry of Health and the WHO Pakistan office has recently released the *National Action Plan for Non-Communicable Disease Prevention, Control and Health Promotion in Pakistan *(NAP-NCD) to achieve national goals for the prevention and control of NCDs [15]. This paper discusses the strengths and limitations of this initiative and highlights the value that such partnership arrangements bring in facilitating the missions and mandates of various partners.

## Merits

The present exercise is the first opportunity to mount a truly 'national plan of action' aimed at preventing and controlling NCDs with the Governments' commitment to NCD prevention as a priority and to enlist a broad range in inputs from within Pakistan for addressing a challenging issue. The NAP-NCD outlines a concerted and comprehensive approach; one that incorporates both policies and actions. It is set within a long-term and life-course perspective and calls for an institutional, community and public policy level change. It has been designed to overcome the tendency to rely on a disjointed set of small scale projects, factoring integration at six levels: grouping NCDs so that they can be targeted through a set of actions, harmonizing actions, integrating actions with existing public health systems, incorporating contemporary evidence-based concepts, combining prevention and health promotion and harnessing the potential within partnerships.

### Disease domain integration

the term NCDs is technically reserved for a group of preventable diseases that are linked by common risk factors: cardiovascular diseases, some chronic lung conditions, cancer and diabetes fall within this category. However NAP-NCD also includes injuries and mental health within this framework as country requirements necessitate that these be addressed within a combined strategic framework through synchronized public health measures. There are many common grounds for combining public health actions to address these diseases.

### Action level integration

**Figure 1:**
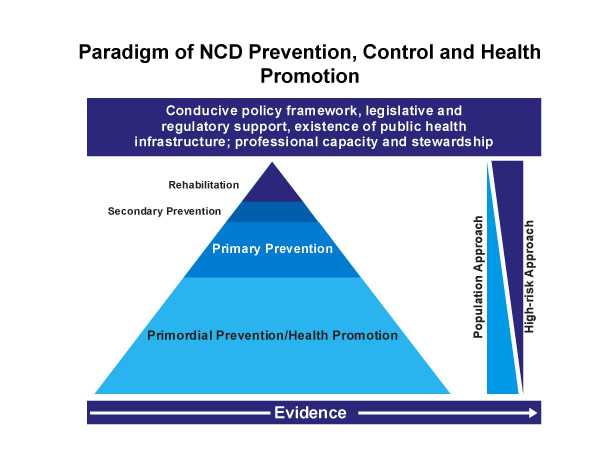
Paradigm for NCD prevention, control and health promotion

the NAP-NCD delivers an Integrated Framework for Action (IFA) [16]; this is modelled to impact a set of indicators through the combination of actions across the range of NCDs in tandem with rigorous formative research. The IFA emanates from the concept highlighted in Fig 1; within this framework, it encompasses two sets of strategies; those that are common across the entire range of NCDs and others that are specific to each NCD domain. The first strategy includes a behavioural change communication strategy, reorientation of health services strategy and surveillance, while the second pertains to legislative and regulatory matters.

### Systems level integration

the approach adopted horizontally integrates NCD prevention with existing public health and social welfare infrastructure. It thus contributes to strengthening of the pubic health configuration and reorients health services to a more preventive orientation.

### Integration of concepts

NAP-NCD packages several contemporary and novel approaches. The population approach includes a behavioural research and social marketing-guided communication strategy and active role of local opinion leaders and educational institutions. Reorientation of health services includes scaling up of professional capacity and basic infrastructure in health care facilities and ensuring availability of, and access to, essential drugs at all levels of health care. The IFA packages a common population surveillance mechanism for all NCD's (with the exception of cancer). The model includes population surveillance of common risk factors and combines a module on population surveillance of injuries and mental health. The model has also been adapted for program evaluation.

### Combining prevention and health promotion

prevention is concerned with avoiding diseases whereas health promotion is about improving health and wellbeing. Both approaches are overlapping and complimentary and can be present in the same programme with similar activities and hold different meanings for two groups of targeted populations with different results. The public health approach to NCDs offers one of the best opportunities to combine prevention and health promotion to improve multiple positive outcomes; an approach NAP-NCD has capitalized on.

### Public-private partnership dimension

this initiative created a mechanism for visible involvement and participation of relevant ministries, educational institutions, NGOs and leadership foci at a national consultation level and created avenues for their participation in the process that led to its development. In addition, all the key elements and advantages that stand to be gained from comprehensive grouping and maximizing on partnerships have been built upon: integration with the existing health system, inter-sectoral and intra-health-sector collaborations, linkage with the national health policy and partnerships with the private sector. NAP-NCD recognizes the scope of partnerships in public health activities and outlines a scope of interventions that are built on shared responsibility, allowing for agencies to participate according to their own missions, mandates, interests and resources. NAP-NCD fosters partnerships and interface arrangements between the public and private sectors so that the federal government is not solely responsible for getting these programmes out to the communities, but can rely on groups and national organizations that have complementary mandates. These partnerships are in harmony with national health priorities, complement state initiatives and are optimally integrated with national health systems.

## Value to participating agencies

### Ministry of Health

Reproductive health and communicable disease prevention and control have traditionally been priority areas for the Ministry of Health. Prevention and control of NCDs did not previously feature as part of the National Health Policy of 2001. There were therefore no specific programs in the national and provincial health departments and no budgetary line for NCD prevention up until the signing of the agreement, which lay the terms of reference for developing NAP-NCD [17]. By leveraging on the technical strengths of a private sector partner, the MOH was able to acquire a scientifically sound plan incorporating broad-based consensus. By adopting this Plan, the MoH has included NCD prevention and control as part of its policy framework. The Federal Government has also shown commitment to implement the plan. Budgetary allocations have been made from the Ministry of Health's existing resources for its first phase of implementation [18]; these will support the establishment of a surveillance system and a behavioural change communication campaign through the media; in addition a training program has been introduced into the work-plan of Lady Health Workers (LHWs), Pakistan's field force of health care providers at the grass roots level in 17 districts targeting a population of approximately 10 million. Heartfile has previously pilot tested this approach in partnership with the MoH [19]. Since Heartfile has the major participatory role in implementing these activities; this approach allows the government to fulfil a policy obligation to include the private sector in national programs outside of a 'contractual' mode. It therefore serves as an empirical basis for health sector reform in the area of public-private collaboration. Overall funding for prevention and health promotion in the national health budget has been increased.

The next stage of implementation of NAP-NCD includes reorientation of health services and a comprehensive school health program. A proposal is already in the funding pipeline to seek additional resources from the governments' development budget and from donor sources.

### World Health Organization

As part of its global mandate, WHO provides technical support to 'priority national programs' through its Joint Government/WHO Program Review Mission (JPRM) program; this is a regular biannual budgetary line. In addition, 'extra-budgetary' resources are provided for 'WHO priority programs' such as the polio campaign. In the year 2003, US $ 843 million were allocated for 192 countries under the former and 1.4 billion as part of the latter [20].

For the year 2001–2003, Pakistan was allocated a budget of US $ 20 million [21]. However by convention, these resources have always been used by public sector institutes and health professionals with public sector affiliations. Within the context of the implementation of the first phase of NAP-NCD, for the first time in Pakistan, the JPRM 2004–05 has made allocations to support activities which are being implemented by an NGO albeit in an official relationship with the MoH [22]. WHO will therefore gain experience in working in a country model in which the private sector can be supported through the JPRM resources.

### Heartfile

Heartfile has been planning and implementing national media campaigns and community-based projects for cardiovascular disease prevention incorporating social marking approaches [23,24]. Although pilot activities have previously been conducted in partnership with the MoH; the NGO activities were previously not integrated with national programs. By partnering in this program, NGOs activities will contribute to the country's National Plans within the framework of priorities set by broad-based national consensus; will be implemented through existing structures and monitored through one evaluation mechanism. Its activities will therefore contribute to achieving national goals.

Currently, the NGO draws support from donor funds through 'project aid'. In future, the NGOs funding is likely to be compromised with shifting donor focus on 'programme aid', as part of which, donors provide funds through national budgets. Partnering in this program therefore contributes to sustainability of the NGO as this provides a mechanism for sustained funding.

## Limitations

The ingredients in this public health strategy are sound; however there are several limitations of this approach. Firstly, it needs to be supported by a clear, strong and sustained political commitment; secondly, the successful implementation of this plan requires the setting up of appropriate infrastructure and public health workforce with adequate capacity for core public health functions. This has implications for the need to build capacity and related infrastructure as a parallel process.

The public-private partnership dimension of this plan emanates from within the overall 'development policy framework', which encourages private-sector participation in state activities. However it does have its own challenges. This experience therefore presents a clear imperative for addressing ethical, methodological, accountability, sustainability and governance issues in public-private and other multi-stakeholder arrangements [25-27].

## Implications for generalisability

Useful lessons can be learnt from this experience both by developing countries and low resource settings that have NCD programs running and others that do not.

Most developing countries have limited capacity for NCD prevention and control [28]. There is limited experience with building 'integrated models' and 'partnerships' for NCD prevention suited to low resource situations. The Action Plan therefore serves as an empirical basis for an integrated approach to NCDs on one hand, and an experimental basis of health sector reform in the area of public-private collaboration on the other.

This example is also relevant to NGOs in other developing countries, who receive financial support from donors through project aid as it serves as an empirical basis for the integration of NGO activities with national plans and goals.

## Evaluation

The desired impact of this intervention is positive change in population health behaviours; therefore its ultimate success can be judged by changes in population outcomes, which can only be assessed over a period of time. However the process evaluation framework of the Action Plan outlined in the IFA is modelled to access how the program achieves its effects and includes the evaluation resource inputs, description of activities and intermediate outcomes. A review of the Logical Framework Analysis of the Action Plan and the IFA has shown progress against many of the process indicators [29].

## Conclusions

Notwithstanding that there are several limitations of this strategy, it does provide the empirical basis for an integrated response to NCD prevention and health promotion in a developing country setting. The IFA is an innovative tool, which helps to set country targets and defines integrated actions to meet those targets. However future efforts must also seek to integrate strategies with communicable disease control, particularly in areas where a life course approach is pursued; this will enable the development of sustainable public health systems for all disease. It is hoped that the findings from this program can be a basis for policy change.
